# Artificial Intelligence Prognostic Models for Healthcare‐Associated Infection Prediction in Adult Intensive Care Units: A Systematic Review Protocol

**DOI:** 10.1002/hsr2.73335

**Published:** 2026-10-05

**Authors:** Antonio Sciurti, Giuseppe Migliara, Leonardo Maria Siena, Giulia Bonanno, Carolina Marzuillo, Paolo Villari, Valentina Baccolini

**Affiliations:** ^1^ Department of Public Health and Infectious Diseases Sapienza University of Rome Rome Italy; ^2^ Department of Life Sciences, Health, and Health Professions Link Campus University Rome Italy; ^3^ UniCamillus ‐ Saint Camillus International University of Health Sciences

**Keywords:** artificial intelligence, clinical prediction models, healthcare‐associated infections, machine learning, prognosis

## Abstract

**Background and Aims:**

Despite widespread Infection Prevention and Control (IPC) measures, Healthcare‐Associated Infections (HAIs) remain a major concern in Intensive Care Units (ICUs), leading to increased mortality, prolonged hospital stays, and additional healthcare costs. Prognostic prediction models support identification of patients at high infection risk, and Machine Learning and Artificial Intelligence (ML/AI) methods offer the potential to improve such predictions. However, no systematic review has specifically examined ML/AI‐based prognostic models for HAI prediction in adult ICU populations. This review aims to identify ML/AI‐based prognostic models for estimating the HAI risk in adult ICU‐patients, investigate their methodological characteristics, risk of bias and reporting quality.

**Methods:**

A systematic search will be conducted in PubMed, Scopus, and Web of Science to identify studies developing and/or validating ML/AI‐based prognostic models of HAI prediction in adult ICU‐admitted patients. Any study developing or evaluating supervised ML/AI‐based models for the prediction of any HAI occurring after ICU admission will be included. Three reviewers will independently screen articles, extract data, and assess risk of bias using the Risk Of Bias ASsessment Tool for prediction models using Artificial Intelligence (PROBAST + AI). Extracted information will include study characteristics, patients and setting, predictors, sample size, missing data, analysis methods, performance, and model presentation and interpretation. A narrative synthesis will be conducted for all included studies. Finally, the SPIN‐Prediction Models (SPIN‐PM) framework will be used to assess quality of reporting. This systematic review protocol was registered on the Open Science Framework (osf. io/bzqfp).

**Conclusion:**

This systematic review's findings on methodological aspects, risk of bias, and applicability of ML/AI‐based prognostic models for HAI prediction in adult ICU patients will help inform researchers, clinicians, and IPC decision‐makers about these models' reliability and readiness for clinical use.

## 1 Introduction

Healthcare‐associated infections (HAIs) are a major concern in modern healthcare settings, particularly within intensive care units (ICUs), with critically ill patients being especially vulnerable due to the frequent use of invasive procedures and the close, continuous contact with healthcare personnel [1]. Although Infection Prevention and Control (IPC) measures—such as hand hygiene protocols, antibiotic stewardship programmes, and infection prevention bundles—are widely implemented [2], HAIs continue to occur most frequently in ICUs [3]. These infections lead to prolonged hospital stays, increased mortality, and substantial financial costs to healthcare systems [4, 5].

Consequently, identifying patients at high risk of developing an HAI during their ICU stay is essential for guiding timely and targeted preventive interventions. To this end, prognostic clinical prediction models are commonly employed to estimate an individual's probability of developing a particular health outcome in the future [6]. In the ICU setting, several existing prognostic models originally designed for mortality prediction, such as severity scoring systems (e.g., SAPS II, APACHE II, SOFA) [7], have been evaluated to estimate HAI risk, but their performance has been inconsistent, reflecting the complex interplay of factors that influence infection risk in this setting [8, 9]. In contrast, by capturing intricate, non‐linear relationships within high‐dimensional clinical data, Machine Learning and Artificial Intelligence (ML/AI) methods are claimed to offer the potential to overcome limitations of traditional modelling approaches and, ultimately, to make more accurate risk predictions [10]. As a result, their application in clinical prediction has expanded rapidly across various healthcare areas, including critical care [11, 12].

However, previous systematic reviews have mainly offered broad overviews of ML/AI applications in HAI surveillance and prevention across different settings [13, 14, 15], often without clearly distinguishing prognostic from diagnostic models (i.e., models that estimate a patient risk of having an infection at the current moment) [16], or focusing mostly on diagnostic models for specific infections such as sepsis or ventilator‐associated pneumonia (VAP) [17, 18]. To our knowledge, no systematic review has synthesised evidence on ML/AI‐based prognostic models for estimating the risk of HAIs among adult ICU‐hospitalised patients. In addition, limited attention has been given to the reporting quality and methodological aspects of studies developing or evaluating ML/AI models in this context, despite recent evidence indicating that ML/AI‐based prediction studies are frequently affected by poor reporting, high risk of bias and limited applicability in practice [19, 20, 21].

To address these gaps, this systematic review aims to (i) identify studies developing or evaluating ML/AI‐based prognostic models for estimating the risk of any HAI in adult patients admitted to the ICU, (ii) summarise the methodologies used to develop and evaluate them, (iii) assess the applicability, risk of bias and reporting quality of these models.

## 2 Methods

We followed the Preferred Reporting Items for Systematic Review and Meta‐Analysis Protocols (PRISMA‐P) [22] (Supporting Table 1) checklist to develop and report this systematic review's protocol. This protocol has been registered on the Open Science Framework (OSF) platform (osf.io/bzqfp), where any major amendment will be documented. The International Prospective Register of Systematic Reviews (PROSPERO) was initially considered for registration, but its usage is currently limited to systematic reviews of health intervention studies.

The CHecklist for critical Appraisal and data extraction for systematic Reviews of prediction Modelling Studies (CHARMS) was used to formulate the systematic review question and guide the review design [23]. Specifically, we adopted the Population, Index models, Comparator, Outcome, Timing and Setting (PICOTS) system to frame the review question and study eligibility criteria [24].

The systematic review will be reported according to the Transparent Reporting of multivariable prediction models for Individual Prognosis Or Diagnosis checklist for Systematic Reviews and Meta‐Analyses (TRIPOD‐SRMA) guidance [25].

### 2.1 Eligibility Criteria

Based on the PICOTS criteria (Table 1), we will include any study that:

i. includes patients of 18 years or older only as the study population;
ii. describes the development and/or evaluation of at least one multivariable prognostic prediction model for HAIs, based on supervised ML/AI methods. In accordance with the guidance for prediction model research [6], we define a prognostic prediction model as a multivariable model, that includes two or more predictors, aimed to predict a future event at the individual patient level, i.e. any HAI after admission to the ICU. We will consider any model that uses nontraditional statistical modelling approaches as a supervised AI/ML‐based model. These methods are different from unpenalized generalized linear models and include, for example, tree‐based methods, support vector machines, neural networks (including deep learning), and other ensemble techniques, i.e., methods that combine predictions from multiple models. In brief, supervised AI/ML methods are used to develop models on existing observations (e.g. patients), including their characteristics and known outcomes, so that they can predict outcomes for new observations with the same set of characteristics and potentially unknown outcomes.
iii. has the occurrence of HAIs of any type and definition (e.g., bloodstream infection [BSI], VAP) as the outcome to be predicted at the individual patient level;
iv. reports the model prediction of HAIs at least 48 h after admission, at least 48 h after a specific ICU event (e.g., after invasive device insertion), or at least 48 h after any time point in ICU stay (fulfilling the definition of prognostic models, i.e., models for predicting future events);
v. is set exclusively in the ICU or includes patients admitted to the ICU alongside patients admitted to other wards (e.g., emergency departments, general wards), provided that separate ICU‐specific prediction models are developed or evaluated;
vi. employs any study design, both experimental (e.g. randomised controlled trials) and observational (e.g., prospective and retrospective cohort, case‐control and case‐cohort studies).

**Table 1:** PICOTS criteria of the systematic review.

Item	Description
Population	Adult patients, aged 18 or older
Index models	Supervised ML/AI‐based prognostic prediction models
Comparator	Not applicable
Outcome	Any type of HAI
Timing	Model prediction at least 48 h after ICU admission, at least 48 h after a specific ICU event (e.g., after invasive device insertion), or at least 48 h after any time point in ICU stay
Setting	Patients admitted to the ICU

Abbreviations: HAI, Healthcare‐Associated Infection; ICU, Intensive Care Unit; ML/AI, Machine Learning/Artificial Intelligence; PICOTS, Population, Index models, Comparator, Outcome, Timing and Setting.

By contrast, we will exclude any study that:

i. includes a study population that is not exclusively composed of adults (≥ 18 years), i.e., paediatric‐only or mixed‐age populations, where models are not separate for adults;
ii. does not describe the development and/or evaluation of multivariable prognostic prediction models (i.e., studies investigating a single predictor or a set of predictors of the same type – e.g., multiple variables measured by a single sensor) and that are not based on ML/AI methods (e.g., unpenalized generalized linear models) and that use ML/AI methods for predictor selection only;
iii. has outcomes to be predicted different from HAIs only (e.g., colonization, antimicrobial resistance acquisition, ICU‐mortality) or predicts outcomes at an aggregate level (e.g., at the ICU level or hospital level) instead of the individual patient level;
iv. reports models that predict the presence of an HAI at any time from ICU admission, with HAIs being present at same time of prediction or within a time window of 48 h or shorter before infection (diagnostic models, i.e., models for predicting current events);
v. includes patients admitted to wards other than ICUs (e.g., emergency departments, general wards) as the study setting or includes patients admitted to both the ICU and other wards in the same model.

Moreover, we will exclude any article that:

i. is not original research (e.g., reviews, commentaries, editorials, letters‐to‐the‐editor, guidelines, expert opinions, books and book chapters);
ii. is not peer‐reviewed (e.g., pre‐prints, study protocols, educational theses);
iii. is published only as a conference paper or proceeding;
iv. is not reported in English;
v. is not available as a full text.

### 2.2 Information Sources and Search Strategy

A systematic search of the following electronic databases will be conducted to identify eligible studies from their inception: PubMed, Scopus, and Web of Science. The search strategy was built using terms related to (i.) HAIs (e.g., Healthcare Associated Infection, Bloodstream Infection, Catheter‐Related Bloodstream Infection, Ventilator‐Associated Pneumonia, Urinary Tract Infection, Catheter‐Associated Urinary Tract Infection), (ii.) ICU (e.g., Intensive Care Unit, Critical Care Units) and (iii.) ML/AI (e.g., Artificial Intelligence, Machine Learning, Statistical Learning, Supervised Learning, LASSO, ridge, elastic net regression, support vector machine, decision tree, gradient boosting machine, XGBoosting, random forest, neural network). See Supporting Table 2 for the full search strategies. Additionally, the reference lists of all included studies and relevant systematic reviews will be manually screened to identify other potentially eligible studies.

### 2.3 Data Management

All records retrieved through the search strategy across databases will be stored and deduplicated for title and abstract screening using citation management software Zotero (ver. 7.0.27, zotero.org, 2025). Once eligible records are identified, full‐text articles will be retrieved for full‐text screening and data extraction. Data items will then be collected from the included studies using an extraction form in a Microsoft Excel spreadsheet.

### 2.4 Study Selection Process

Three researchers (AS, LMS, GB) will independently screen titles and abstracts to identify records that meet the eligibility criteria. Subsequently, they will independently assess the full texts of potentially eligible articles. In both stages, one researcher (AS) will screen all articles, and the others will jointly review a subset of the same articles to ensure consistency. Any disagreement will be resolved by consensus or consultation with a third reviewer (GM). The selection flow will be presented in a flowchart [25].

### 2.5 Data Collection Process and Items

Information from included studies will be extracted using a data extraction form. Items to be extracted will be informed by the CHARMS [23] and TRIPOD+Artificial Intelligence (TRIPOD + AI) [26] checklists. Information about the following domains will be extracted:

i. General information (first author, publication year, country, study design, source of data, type of prediction model study, adherence to reporting guidelines);
ii. Patients and setting (patient eligibility criteria, patient description, study dates, ICU type);
iii. Outcome (type and definition of HAI, same outcome definition used for all patients, blinded outcome assessment, whether candidate predictors were part of the outcome definition, time of occurrence of the outcome, summary of follow‐up duration);
iv. Predictors (number and type of candidate predictors, timing of candidate predictor definition/measurement, blinded candidate predictor assessment, rationale/methods to handle continuous candidate predictors);
v. Sample size (number of patients, number and proportion of events, number of events per candidate predictor, sample size justification);
vi. Missing data (handling and presentation of missing data);
vii. Analysis methods (supervised ML/AI method, method for selection of predictors before modelling and during modelling, methods for dimensionality reduction, methods to handle class imbalance, hyperparameter tuning strategy, method of model evaluation, model adjustment or update after evaluation, methods for calculation of predictions);
viii. Performance (discrimination and classification measures with confidence intervals and cut‐points, calibration measures with confidence intervals, additional presentation of the models);
ix. Model presentation and interpretation (number of final models, number and type of final predictors selected in models, importance of predictors in final models, discussion of model generalizability/transportability, model usability in current care);
x. Open science practices (study protocol availability and registration, data and code availability).

The full data extraction form is available in Supporting Table 3. The data extraction form will be piloted on a set of five studies and revised, if needed. Three reviewers (AS, LMS, GB) will independently extract data from each included study. Data will be extracted from all included studies by one reviewer (AS), while the remaining researchers will jointly extract data from a subset of the same studies for verification (LMS, GB). Any discrepancies in data extraction will be discussed between the reviewers and, if necessary, resolved through consultation with a third reviewer (GM).

### 2.6 Risk of Bias and Applicability Assessment

The risk of bias and applicability of included studies will be assessed using the Prediction model Risk Of Bias ASsessment Tool for prediction models using Artificial Intelligence methods (PROBAST + AI) [27]. Three reviewers (AS, LMS, GB) will independently assess signalling questions across four domains (participants and data sources, predictors, outcomes, and analysis) for studies reporting either model development or model evaluation, or both, to judge the overall concern on quality and applicability of model development, as well as the risk of bias and applicability concerns for model evaluation. One researcher (AS) will assess risk of bias and applicability of individual studies for each study and the other reviewers (LMS, GB) will perform the assessment from a subset of the same studies. Any discrepancies will be resolved through discussion or, if needed, by consultation with a third reviewer (GM).

### 2.7 Data Synthesis

A narrative synthesis of the extracted data will be conducted. Categorical data will be presented as numbers and percentages. Continuous data will be assessed for distribution and described using mean ± standard deviation if normally distributed, or median and interquartile range (25th–75th percentiles) if non‐normally distributed. Risk of bias assessments will be summarized and graphically presented for each PROBAST + AI domain and for overall judgment.

If enough multiple external evaluation studies of the same model are retrieved, we will perform a meta‐analysis of discrimination (e.g., area under the curve, c‐statistic) and calibration estimates (e.g., observed expected [O:E] ratio, calibration slope), using a random effects model to account for potential between‐study heterogeneity, using the I^2^ metric to quantify heterogeneity. Sources of heterogeneity will be explored with subgroup analyses and meta‐regression [24, 28].

All analyses will be performed in R statistical software (ver. 4.5.1, r‐project.org, 2025).

### 2.8 Meta‐Bias Assessment

Meta‐bias will not be formally investigated in this study. We will investigate potential spin practices in the included studies. Spin is defined as any reporting practice that leads to a misleading interpretation or overestimation of transportability of the study findings. Therefore, we will use the SPIN‐Prediction Models (SPIN‐PM) framework to detect relevant spin practices and their facilitators in the included studies [29].

### 2.9 Confidence in Cumulative Evidence

If enough external validation studies of the same model are retrieved, we will assess the certainty of the body of evidence according to the Grading of Recommendations, Assessment, Development and Evaluation (GRADE) guidance for prognostic model evaluation studies [30, 31]. Specifically, we will use a c‐statistic threshold of 0.7 for discrimination, representing clinical intuition [32], and an O:E ratio between 0.9 and 1.1 for calibration [30, 31].

## 3 Conclusion

By identifying studies developing and/or evaluating supervised ML/AI‐based prognostic models for predicting HAIs in adult patients admitted to the ICU, this systematic review will provide an overview of the methodological conduct, risk of bias, and applicability of these models, thereby informing researchers, clinicians, and IPC decision‐makers about the extent to which these models are ready for deployment or whether further methodological refinements are required before reliable clinical use.

## Author Contributions

**Antonio Sciurti:** conceptualization, methodology, data curation, writing – original draft, writing – review and editing. **Giuseppe Migliara:** writing – review and editing, methodology, data curation. **Leonardo Maria Siena:** writing – review and editing, data curation. **Giulia Bonanno:** data curation, writing – review and editing. **Carolina Marzuillo:** writing – review and editing. **Paolo Villari:** writing – review and editing. **Valentina Baccolini:** writing – review and editing, supervision, methodology.

## Funding

The authors have nothing to report.

## Ethics Statement

This study will not require ethical approval as no primary data will be collected. The results of this review will be presented at seminars, conferences and symposia, as well as published in a peer‐reviewed journal.

## Conflicts of Interest

The authors declare no conflicts of interest.

## Author Declaration

All authors have read and approved the final version of the manuscript. The lead author, Antonio Sciurti, had full access to all of the data in this study and takes complete responsibility for the integrity of the data and the accuracy of the data analysis.

## Transparency Statement

The lead author, Antonio Sciurti, confirms that this manuscript presents an honest, accurate, and transparent account of the reported study protocol; that no significant elements have been excluded; and that any deviations from the original study plan (and registration, if applicable) will be fully explained.

## Supporting information


Supporting File

## Data Availability

This protocol and supporting materials are available on the Open Science Framework (osf.io/bzqfp).
