# Supplementary material for: Artificial Intelligence Prognostic Models for Healthcare‐Associated Infection Prediction in Adult Intensive Care Units: A Systematic Review Protocol
Source: Health Sci Rep. 2026 Oct 5;9(10):e73335. doi: 10.1002/hsr2.73335 (PMC13635808; doi:10.1002/hsr2.73335)
Supplement: Supplementary file 1 [file HSR2-9-e73335-s001.docx]

### Supporting Table 1. PRISMA-P checklist.

| **Section/topic** | **#** | **Item** | **Reported**  **(page/line number)** |
| --- | --- | --- | --- |
| **ADMINISTRATIVE INFORMATION** | | |  |
| **Title** |  |  |  |
| Identification | 1a | Identify the report as a protocol of a systematic review | 1-2 |
| Update | 1b | If the protocol is for an update of a previous systematic review, identify as such | NA |
| **Registration** | 2 | If registered, provide the name of the registry (e.g., PROSPERO) and registration number in the Abstract | 49-50 |
| **Authors** |  |  |  |
| Contact | 3a | Provide name, institutional affiliation, and e-mail address of all protocol authors; provide physical mailing address of corresponding author | Page 1 |
| Contributions | 3b | Describe contributions of protocol authors and identify the guarantor of the review | 9-14 |
| **Amendments** | 4 | If the protocol represents an amendment of a previously completed or published protocol, identify as such and list changes; otherwise, state plan for documenting important protocol amendments | NA |
| **Support** |  |  |  |
| Sources | 5a | Indicate sources of financial or other support for the review | 16 |
| Sponsor | 5b | Provide name for the review funder and/or sponsor | NA |
| Role of sponsor/funder | 5c | Describe roles of funder(s), sponsor(s), and/or institution(s), if any, in developing the protocol | NA |
| **INTRODUCTION** |  |  |  |
| **Rationale** | 6 | Describe the rationale for the review in the context of what is already known | 80-90 |
| **Objectives** | 7 | Provide an explicit statement of the question(s) the review will address with reference to participants, interventions, comparators, and outcomes (e.g., PICO) | 91-94 |
| **METHODS** |  |  |  |
| **Eligibility criteria** | 8 | Specify the study characteristics (e.g., PICO, study design, setting, time frame) and report characteristics (e.g., years considered, language, publication status) to be used as criteria for eligibility for the review | 113-162,  Table 1 |
| **Information sources** | 9 | Describe all intended information sources (e.g., electronic databases, contact with study authors, trial registers, or other grey literature sources) with planned dates of coverage | 164-175 |
| **Search strategy** | 10 | Present draft of search strategy to be used for at least one electronic database, including planned limits, such that it could be repeated | Supporting Table 1 |
| **Study records** |  |  |  |
| Data management | 11a | Describe the mechanism(s) that will be used to manage records and data throughout the review | 177-182 |
| Selection process | 11b | State the process that will be used for selecting studies (e.g., two independent reviewers) through each phase of the review (i.e., screening, eligibility, and inclusion in meta-analysis) | 184-190 |
| Data collection process | 11c | Describe planned method of extracting data from reports (e.g., piloting forms, done independently, in duplicate), any processes for obtaining and confirming data from investigators | 219-225 |
| **Data items** | 12 | List and define all variables for which data will be sought (e.g., PICO items, funding sources), any pre-planned data assumptions and simplifications | 192-198, 202-217 |
| **Outcomes and prioritization** | 13 | List and define all outcomes for which data will be sought, including prioritization of main and additional outcomes, with rationale | 199-201 |
| **Risk of bias in individual studies** | 14 | Describe anticipated methods for assessing risk of bias of individual studies, including whether this will be done at the outcome or study level, or both; state how this information will be used in data synthesis | 227-237 |
| **Data synthesis** | 15a | Describe criteria under which study data will be quantitatively synthesized | 254-250 |
|  | 15b | If data are appropriate for quantitative synthesis, describe planned summary measures, methods of handling data, and methods of combining data from studies, including any planned exploration of consistency (e.g., *I* ^2^, Kendall’s tau) | 247-251 |
|  | 15c | Describe any proposed additional analyses (e.g., sensitivity or subgroup analyses, meta-regression) | 249 |
|  | 15d | If quantitative synthesis is not appropriate, describe the type of summary planned | 240-244 |
| **Meta-bias(es)** | 16 | Specify any planned assessment of meta-bias(es) (e.g., publication bias across studies, selective reporting within studies) | 252-257 |
| **Confidence in cumulative evidence** | 17 | Describe how the strength of the body of evidence will be assessed (e.g., GRADE) | 259-260 |

GRADE: Grading of Recommendations Assessment, Development, and Evaluation; NA: Not applicable; PICO: Patient, Intervention, Comparator and Outcome; PRISMA-P: Preferred Reporting Items for Systematic Review and Meta-Analysis Protocols; PROSPERO: International Prospective Register of Systematic Reviews.

### Supporting Table 2. Full search strategies by database.

| **Database** | **Search query** |
| --- | --- |
| **PubMed** | (("healthcare associated infection"[tw] OR "healthcare associated infections"[tw] OR "healthcare-associated infection "[tw] OR "healthcare-associated infections"[tw] OR "healthcare related infection"[tw] OR "healthcare-related infections"[tw] OR "health care associated infection"[tw] OR "health care associated infections"[tw] OR "health care-associated infection "[tw] OR "health care-associated infections"[tw] OR "health care related infection"[tw] OR "health care-related infections"[tw] OR "health-care associated infection"[tw] OR "health-care associated infections"[tw] OR "health-care-associated infection"[tw] OR "health-care-associated infections"[tw] OR "health-care related infection"[tw] OR "health-care-related infections"[tw] OR "hospital acquired infection"[tw] OR "hospital acquired infections"[tw] OR "hospital-acquired infection"[tw] OR "hospital-acquired infections"[tw] OR "nosocomial infection"[tw] OR "nosocomial infections"[tw] OR "cross infection"[tw] OR "cross infections"[tw] OR "cross-infection"[tw] OR "cross-infections"[tw] OR "HAI"[tw] OR "HAIs"[tw] OR "HCAI"[tw] OR "HCAIs"[tw] OR "intensive care unit-acquired infection"[tw] OR "intensive care unit-acquired infections"[tw] OR "ICU-acquired infection"[tw] OR "ICU-acquired infections"[tw] OR "intensive care unit-acquired infection"[tw] OR "intensive care unit-acquired infections"[tw] OR "ICU-acquired infection"[tw] OR "ICU-acquired infections"[tw] OR "intensive care unit-associated infection"[tw] OR "intensive care unit-associated infections"[tw] OR "ICU-associated infection"[tw] OR "ICU-associated infections"[tw] OR "intensive care unit-associated infection"[tw] OR "intensive care unit-associated infections"[tw] OR "ICU-associated infection"[tw] OR "ICU-associated infections"[tw] OR "intensive care unit-related infection"[tw] OR "intensive care unit-related infections"[tw] OR "ICU-related infection"[tw] OR "ICU-related infections"[tw] OR "intensive care unit-related infection"[tw] OR "intensive care unit-related infections"[tw] OR "ICU-related infection"[tw] OR "ICU-related infections"[tw] OR "device associated infection"[tw] OR "device associated infections"[tw] OR "device-associated infection"[tw] OR "device-associated infections"[tw] OR "device related infection"[tw] OR "device related infections"[tw] OR "device-related infection"[tw] OR "device-related infections"[tw] OR **"Cross Infection"[mh] OR** "bloodstream infection"[tw] OR "bloodstream infections"[tw] OR "blood-stream infection"[tw] OR "blood-stream infections"[tw] OR "sepsis"[tw] OR "sepses"[tw] OR "bacteremia"[tw] OR "bacteraemia"[tw] OR "septicemia"[tw] OR "septicaemia"[tw] OR "bacteremias"[tw] OR "bacteraemias"[tw] OR "septicemias"[tw] OR "septicaemias"[tw] OR "BSI"[tw] OR "BSIs"[tw] OR "catheter related infection"[tw] OR "catheter related infections"[tw] OR "catheter-related infection"[tw] OR "catheter-related infections"[tw] OR "CRI"[tw] OR "CRIs"[tw] OR "catheter related bloodstream infection"[tw] OR "catheter related bloodstream infections"[tw] OR "catheter-related bloodstream infection"[tw] OR "catheter-related bloodstream infections"[tw] OR "catheter related blood-stream infection"[tw] OR "catheter related blood-stream infections"[tw] OR "catheter-related blood-stream infection"[tw] OR "catheter-related blood-stream infections"[tw] OR "CRBSI"[tw] OR "CRBSIs"[tw] OR "CR-BSI"[tw] OR "CR-BSIs"[tw] OR "central line associated infection"[tw] OR "central line associated infections"[tw] OR "central line-associated infection"[tw] OR "central line-associated infections"[tw] OR "CLABSI"[tw] OR "CLABSIs"[tw] OR "CLA-BSI"[tw] OR "CLA-BSIs"[tw] OR **"Sepsis"[mh] OR** "pneumonia"[tw] OR "pneumonias"[tw] OR "PNEU"[tw] OR "PNEUs"[tw] OR "lower respiratory tract infections"[tw] OR "lower respiratory tract infection"[tw] OR "LRTI"[tw] OR "LRTIs"[tw] OR "intubation associated pneumonia"[tw] OR "intubation associated pneumonias"[tw] OR "intubation-associated pneumonia"[tw] OR "intubation associated pneumonias"[tw] OR "IAP"[tw] OR "IAPs"[tw] OR "ventilator associated pneumonia"[tw] OR "ventilator associated pneumonias"[tw] OR "ventilator-associated pneumonia"[tw] OR "ventilator-associated pneumonias"[tw] OR "ventilation associated pneumonia"[tw] OR "ventilation associated pneumonias"[tw] OR "ventilation-associated pneumonia"[tw] OR "ventilation-associated pneumonias"[tw] OR "ventilator associated event"[tw] OR "ventilator associated events"[tw] OR "ventilator-associated event"[tw] OR "ventilator-associated events"[tw] OR "ventilation associated event"[tw] OR "ventilation associated events"[tw] OR "ventilation-associated event"[tw] OR "ventilation-associated events"[tw] OR "VAP"[tw] OR "VAPs"[tw] OR "VAE"[tw] OR "VAEs"[tw] OR **"Healthcare-Associated Pneumonia"[mh] OR** "urinary tract infection"[tw] OR "urinary tract infections"[tw] OR "UTI"[tw] OR "UTIs"[tw] OR "catheter-associated infection"[tw] OR "catheter-associated infections"[tw] OR "catheter associated infection"[tw] OR "catheter associated infections"[tw] OR "CAUTI"[tw] OR "CAUTIs"[tw] OR "CA-UTI"[tw] OR "CA-UTIs"[tw] OR **"Urinary Tract Infections"[mh] OR** "surgical site infection"[tw] OR "surgical site infections"[tw] OR "surgical wound infection"[tw] OR "surgical wound infections"[tw] OR "SSI"[tw] OR "SSIs"[tw] OR **"Surgical Wound Infection"[mh]**)) **AND** (("care unit"[tw] OR "care units"[tw] OR "intensive care"[tw] OR "intensive-care"[tw] OR "critical care"[tw] OR "critical-care"[tw] OR "ICU"[tw] OR "ICUs"[tw] OR **"Intensive Care Units"[mh] OR "Critical Care"[mh]**)) **AND** (("artificial intelligence"[tw] OR "machine learning"[tw] OR "statistical learning"[tw] OR "supervised learning"[tw] OR "unsupervised learning"[tw] OR "clustering"[tw] OR **"Artificial Intelligence"[mh] OR "Machine Learning"[mh] OR** "ridge"[tw] OR "LASSO"[tw] OR "elastic net"[tw] OR "elastic-net"[tw] OR "support vector machine"[tw] OR "support vector machines"[tw] OR "SVM"[tw] OR "SVMs"[tw] OR "nearest neighbor"[tw] OR "nearest neighbour"[tw] OR "k-nn"[tw] OR "k nn"[tw] OR "naïve Bayes"[tw] OR "decision tree"[tw] OR "decision trees"[tw] OR "classification tree"[tw] OR "classification trees"[tw] OR "regression tree"[tw] OR "regression trees"[tw] OR "classification and regression tree"[tw] OR "classification and regression tree"[tw] OR "CART"[tw] OR "conditional inference decision"[tw] OR "CIDT"[tw] OR "bagging"[tw] OR "boosting"[tw] OR "gradient boosting"[tw] OR "gradient-boosting"[tw] OR "GBM"[tw] OR "AdaBoost"[tw] OR "AdaBoosting"[tw] OR "XGBoost"[tw] OR "XGBoosting"[tw] OR "XGB"[tw] OR "CatBoost"[tw] OR "CatBoosting"[tw] OR "LightGBM"[tw] OR "logitboost"[tw] OR "logitboosting"[tw] OR "RUSBoost"[tw] OR "RUSBoosting"[tw] OR "extremely randomized trees"[tw] OR "extremely randomised trees"[tw] OR "random forest"[tw] OR "random forests"[tw] OR "perceptron"[tw] OR "perceptrons"[tw] OR "neural network"[tw] OR "neural networks"[tw] OR "artificial neural network"[tw] OR "artificial neural networks"[tw] OR "ANN"[tw] OR "ANNs"[tw] OR "recurrent neural network"[tw] OR "recurrent neural networks"[tw] OR "RNN"[tw] OR "RNNs"[tw] OR "long-short term memory"[tw] OR "LSTM"[tw] OR "gated recurrent unit"[tw] OR "GRU"[tw] OR "convolutional neural network"[tw] OR "convolutional neural networks"[tw] OR "CNN"[tw] OR "CNNs"[tw] OR "densely connected convolutional network"[tw] OR "densely connected convolutional networks"[tw] OR "denseNet"[tw] OR "autoencoder"[tw] OR "autoencoders"[tw] OR "transformer"[tw] OR "transformers"[tw] OR "language model"[tw] OR "language models"[tw] OR "LLM"[tw] OR "LLMs"[tw] OR **"Neural Networks, Computer"[mh] OR "Deep Learning"[mh]** OR "ensembles"[tw] OR "ensemble learner"[tw] OR "ensemble learners"[tw] OR "ensemble model"[tw] OR "ensemble models"[tw] OR "superlearner"[tw] OR "superlearners"[tw] OR "super-learner"[tw] OR "super-learners"[tw] OR "super learner"[tw] OR "super learners"[tw] OR "meta-learner"[tw] OR "meta-learners"[tw] OR "meta learner"[tw] OR "meta learners"[tw])) |
| **Scopus** | TITLE-ABS-KEY("healthcare associated infection" OR "healthcare associated infections" OR "healthcare-associated infection" OR "healthcare-associated infections" OR "healthcare related infection" OR "healthcare-related infections" OR "health care associated infection" OR "health care associated infections" OR "health care-associated infection" OR "health care-associated infections" OR "health care related infection" OR "health care-related infections" OR "health-care associated infection" OR "health-care associated infections" OR "health-care-associated infection" OR "health-care-associated infections" OR "health-care related infection" OR "health-care-related infections" OR "hospital acquired infection" OR "hospital acquired infections" OR "hospital-acquired infection" OR "hospital-acquired infections" OR "nosocomial infection" OR "nosocomial infections" OR "cross infection" OR "cross infections" OR "cross-infection" OR "cross-infections" OR "HAI" OR "HAIs" OR "HCAI" OR "HCAIs" OR "intensive care unit-acquired infection" OR "intensive care unit-acquired infections" OR "ICU-acquired infection" OR "ICU-acquired infections" OR "intensive care unit-acquired infection" OR "intensive care unit-acquired infections" OR "ICU-acquired infection" OR "ICU-acquired infections" OR "intensive care unit-associated infection" OR "intensive care unit-associated infections" OR "ICU-associated infection" OR "ICU-associated infections" OR "intensive care unit-associated infection" OR "intensive care unit-associated infections" OR "ICU-associated infection" OR "ICU-associated infections" OR "intensive care unit-related infection" OR "intensive care unit-related infections" OR "ICU-related infection" OR "ICU-related infections" OR "intensive care unit-related infection" OR "intensive care unit-related infections" OR "ICU-related infection" OR "ICU-related infections" OR "device associated infection" OR "device associated infections" OR "device-associated infection" OR "device-associated infections" OR "device related infection" OR "device related infections" OR "device-related infection" OR "device-related infections" OR "bloodstream infection" OR "bloodstream infections" OR "blood-stream infection" OR "blood-stream infections" OR "sepsis" OR "sepses" OR "bacteremia" OR "bacteraemia" OR "septicemia" OR "septicaemia" OR "bacteremias" OR "bacteraemias" OR "septicemias" OR "septicaemias" OR "BSI" OR "BSIs" OR "catheter related infection" OR "catheter related infections" OR "catheter-related infection" OR "catheter-related infections" OR "CRI" OR "CRIs" OR "catheter related bloodstream infection" OR "catheter related bloodstream infections" OR "catheter-related bloodstream infection" OR "catheter-related bloodstream infections" OR "catheter related blood-stream infection" OR "catheter related blood-stream infections" OR "catheter-related blood-stream infection" OR "catheter-related blood-stream infections" OR "CRBSI" OR "CRBSIs" OR "CR-BSI" OR "CR-BSIs" OR "central line associated infection" OR "central line associated infections" OR "central line-associated infection" OR "central line-associated infections" OR "CLABSI" OR "CLABSIs" OR "CLA-BSI" OR "CLA-BSIs" OR "pneumonia" OR "pneumonias" OR "PNEU" OR "PNEUs" OR "lower respiratory tract infections" OR "lower respiratory tract infection" OR "LRTI" OR "LRTIs" OR "intubation associated pneumonia" OR "intubation associated pneumonias" OR "intubation-associated pneumonia" OR "intubation associated pneumonias" OR "IAP" OR "IAPs" OR "ventilator associated pneumonia" OR "ventilator associated pneumonias" OR "ventilator-associated pneumonia" OR "ventilator-associated pneumonias" OR "ventilation associated pneumonia" OR "ventilation associated pneumonias" OR "ventilation-associated pneumonia" OR "ventilation-associated pneumonias" OR "ventilator associated event" OR "ventilator associated events" OR "ventilator-associated event" OR "ventilator-associated events" OR "ventilation associated event" OR "ventilation associated events" OR "ventilation-associated event" OR "ventilation-associated events" OR "VAP" OR "VAPs" OR "VAE" OR "VAEs" OR "urinary tract infection" OR "urinary tract infections" OR "UTI" OR "UTIs" OR "catheter-associated infection" OR "catheter-associated infections" OR "catheter associated infection" OR "catheter associated infections" OR "CAUTI" OR "CAUTIs" OR "CA-UTI" OR "CA-UTIs" OR "surgical site infection" OR "surgical site infections" OR "surgical wound infection" OR "surgical wound infections" OR "SSI" OR "SSIs") **AND** TITLE-ABS-KEY("care unit" OR "care units" OR "intensive care" OR "intensive-care" OR "critical care" OR "critical-care" OR "ICU" OR "ICUs") **AND** TITLE-ABS-KEY("artificial intelligence" OR "machine learning" OR "statistical learning" OR "supervised learning" OR "unsupervised learning" OR "clustering" OR "ridge" OR "LASSO" OR "elastic net" OR "elastic-net" OR "support vector machine" OR "support vector machines" OR "SVM" OR "SVMs" OR "nearest neighbor" OR "nearest neighbour" OR "k-nn" OR "k nn" OR "naïve Bayes" OR "decision tree" OR "decision trees" OR "classification tree" OR "classification trees" OR "regression tree" OR "regression trees" OR "classification and regression tree" OR "classification and regression tree" OR "CART" OR "conditional inference decision" OR "CIDT" OR "bagging" OR "boosting" OR "gradient boosting" OR "gradient-boosting" OR "GBM" OR "AdaBoost" OR "AdaBoosting" OR "XGBoost" OR "XGBoosting" OR "XGB" OR "CatBoost" OR "CatBoosting" OR "LightGBM" OR "logitboost" OR "logitboosting" OR "RUSBoost" OR "RUSBoosting" OR "extremely randomized trees" OR "extremely randomised trees" OR "random forest" OR "random forests" OR "perceptron" OR "perceptrons" OR "neural network" OR "neural networks" OR "artificial neural network" OR "artificial neural networks" OR "ANN" OR "ANNs" OR "recurrent neural network" OR "recurrent neural networks" OR "RNN" OR "RNNs" OR "long-short term memory" OR "LSTM" OR "gated recurrent unit" OR "GRU" OR "convolutional neural network" OR "convolutional neural networks" OR "CNN" OR "CNNs" OR "densely connected convolutional network" OR "densely connected convolutional networks" OR "denseNet" OR "autoencoder" OR "autoencoders" OR "transformer" OR "transformers" OR "language model" OR "language models" OR "LLM" OR "LLMs" OR "ensembles" OR "ensemble learner" OR "ensemble learners" OR "ensemble model" OR "ensemble models" OR "superlearner" OR "superlearners" OR "super-learner" OR "super-learners" OR "super learner" OR "super learners" OR "meta-learner" OR "meta-learners" OR "meta learner" OR "meta learners") |
| **Web of Science** | TS=("healthcare associated infection" OR "healthcare associated infections" OR "healthcare-associated infection" OR "healthcare-associated infections" OR "healthcare related infection" OR "healthcare-related infections" OR "health care associated infection" OR "health care associated infections" OR "health care-associated infection" OR "health care-associated infections" OR "health care related infection" OR "health care-related infections" OR "health-care associated infection" OR "health-care associated infections" OR "health-care-associated infection" OR "health-care-associated infections" OR "health-care related infection" OR "health-care-related infections" OR "hospital acquired infection" OR "hospital acquired infections" OR "hospital-acquired infection" OR "hospital-acquired infections" OR "nosocomial infection" OR "nosocomial infections" OR "cross infection" OR "cross infections" OR "cross-infection" OR "cross-infections" OR "HAI" OR "HAIs" OR "HCAI" OR "HCAIs" OR "intensive care unit-acquired infection" OR "intensive care unit-acquired infections" OR "ICU-acquired infection" OR "ICU-acquired infections" OR "intensive care unit-acquired infection" OR "intensive care unit-acquired infections" OR "ICU-acquired infection" OR "ICU-acquired infections" OR "intensive care unit-associated infection" OR "intensive care unit-associated infections" OR "ICU-associated infection" OR "ICU-associated infections" OR "intensive care unit-associated infection" OR "intensive care unit-associated infections" OR "ICU-associated infection" OR "ICU-associated infections" OR "intensive care unit-related infection" OR "intensive care unit-related infections" OR "ICU-related infection" OR "ICU-related infections" OR "intensive care unit-related infection" OR "intensive care unit-related infections" OR "ICU-related infection" OR "ICU-related infections" OR "device associated infection" OR "device associated infections" OR "device-associated infection" OR "device-associated infections" OR "device related infection" OR "device related infections" OR "device-related infection" OR "device-related infections" OR "bloodstream infection" OR "bloodstream infections" OR "blood-stream infection" OR "blood-stream infections" OR "sepsis" OR "sepses" OR "bacteremia" OR "bacteraemia" OR "septicemia" OR "septicaemia" OR "bacteremias" OR "bacteraemias" OR "septicemias" OR "septicaemias" OR "BSI" OR "BSIs" OR "catheter related infection" OR "catheter related infections" OR "catheter-related infection" OR "catheter-related infections" OR "CRI" OR "CRIs" OR "catheter related bloodstream infection" OR "catheter related bloodstream infections" OR "catheter-related bloodstream infection" OR "catheter-related bloodstream infections" OR "catheter related blood-stream infection" OR "catheter related blood-stream infections" OR "catheter-related blood-stream infection" OR "catheter-related blood-stream infections" OR "CRBSI" OR "CRBSIs" OR "CR-BSI" OR "CR-BSIs" OR "central line associated infection" OR "central line associated infections" OR "central line-associated infection" OR "central line-associated infections" OR "CLABSI" OR "CLABSIs" OR "CLA-BSI" OR "CLA-BSIs" OR "pneumonia" OR "pneumonias" OR "PNEU" OR "PNEUs" OR "lower respiratory tract infections" OR "lower respiratory tract infection" OR "LRTI" OR "LRTIs" OR "intubation associated pneumonia" OR "intubation associated pneumonias" OR "intubation-associated pneumonia" OR "intubation associated pneumonias" OR "IAP" OR "IAPs" OR "ventilator associated pneumonia" OR "ventilator associated pneumonias" OR "ventilator-associated pneumonia" OR "ventilator-associated pneumonias" OR "ventilation associated pneumonia" OR "ventilation associated pneumonias" OR "ventilation-associated pneumonia" OR "ventilation-associated pneumonias" OR "ventilator associated event" OR "ventilator associated events" OR "ventilator-associated event" OR "ventilator-associated events" OR "ventilation associated event" OR "ventilation associated events" OR "ventilation-associated event" OR "ventilation-associated events" OR "VAP" OR "VAPs" OR "VAE" OR "VAEs" OR "urinary tract infection" OR "urinary tract infections" OR "UTI" OR "UTIs" OR "catheter-associated infection" OR "catheter-associated infections" OR "catheter associated infection" OR "catheter associated infections" OR "CAUTI" OR "CAUTIs" OR "CA-UTI" OR "CA-UTIs" OR "surgical site infection" OR "surgical site infections" OR "surgical wound infection" OR "surgical wound infections" OR "SSI" OR "SSIs") **AND** TS=("care unit" OR "care units" OR "intensive care" OR "intensive-care" OR "critical care" OR "critical-care" OR "ICU" OR "ICUs") **AND** TS=("artificial intelligence" OR "machine learning" OR "statistical learning" OR "supervised learning" OR "unsupervised learning" OR "clustering" OR "ridge" OR "LASSO" OR "elastic net" OR "elastic-net" OR "support vector machine" OR "support vector machines" OR "SVM" OR "SVMs" OR "nearest neighbor" OR "nearest neighbour" OR "k-nn" OR "k nn" OR "naïve Bayes" OR "decision tree" OR "decision trees" OR "classification tree" OR "classification trees" OR "regression tree" OR "regression trees" OR "classification and regression tree" OR "classification and regression tree" OR "CART" OR "conditional inference decision" OR "CIDT" OR "bagging" OR "boosting" OR "gradient boosting" OR "gradient-boosting" OR "GBM" OR "AdaBoost" OR "AdaBoosting" OR "XGBoost" OR "XGBoosting" OR "XGB" OR "CatBoost" OR "CatBoosting" OR "LightGBM" OR "logitboost" OR "logitboosting" OR "RUSBoost" OR "RUSBoosting" OR "extremely randomized trees" OR "extremely randomised trees" OR "random forest" OR "random forests" OR "perceptron" OR "perceptrons" OR "neural network" OR "neural networks" OR "artificial neural network" OR "artificial neural networks" OR "ANN" OR "ANNs" OR "recurrent neural network" OR "recurrent neural networks" OR "RNN" OR "RNNs" OR "long-short term memory" OR "LSTM" OR "gated recurrent unit" OR "GRU" OR "convolutional neural network" OR "convolutional neural networks" OR "CNN" OR "CNNs" OR "densely connected convolutional network" OR "densely connected convolutional networks" OR "denseNet" OR "autoencoder" OR "autoencoders" OR "transformer" OR "transformers" OR "language model" OR "language models" OR "LLM" OR "LLMs" OR "ensembles" OR "ensemble learner" OR "ensemble learners" OR "ensemble model" OR "ensemble models" OR "superlearner" OR "superlearners" OR "super-learner" OR "super-learners" OR "super learner" OR "super learners" OR "meta-learner" OR "meta-learners" OR "meta learner" OR "meta learners") |

### Supporting Table 3. Data extraction form.

| **Domain** | **Item** |
| --- | --- |
| **General information** | First author |
|  | Publication year |
|  | Country |
|  | Study design (i.e., experimental [randomised controlled trials], observational [prospective, retrospective cohort, case-control, case-cohort studies]) |
|  | Source of data (e.g., ad hoc collection, EMR, registry, administrative claims) |
|  | Type of prediction model study (i.e., model development, model evaluation, or both) |
|  | Adherence to reporting guidelines (e.g., CONSORT, STARD, STROBE, TRIPOD) |
| **Patients and setting** | Patient eligibility criteria (patient inclusion and exclusion criteria) |
|  | Patient description (e.g., patient characteristics in development and evaluation sets) |
|  | Study dates (i.e., start and end dates of patient follow-up) |
|  | Type of ICU (e.g., general, neurological, burn, surgical, trauma intensive care unit) |
| **Outcome** | Type of HAI (e.g., any HAI, BSI, pneumonia, UTI) |
|  | HAI definition (e.g., definition by NHSN, ECDC) |
|  | Same outcome definition used for all patients |
|  | Blinded outcome assessment (outcome assessed without knowledge of candidate predictors) |
|  | Candidate predictors part of the outcome (e.g., in panel or consensus diagnosis) |
|  | Time of occurrence of the outcome |
|  | Summary of follow-up duration |
| **Predictors** | Number of candidate predictors |
|  | Type of candidate predictors (e.g., demographics, clinical history, physical examination, laboratory testing, imaging, ICU severity scores) |
|  | Timing of candidate predictor definition/measurement (e.g., at admission or in defined time-window after admission) |
|  | Blinded candidate predictor assessment (candidate predictors assessed without knowledge of outcome) |
|  | Rationale/methods to handle continuous candidate predictors (e.g., non-linear transformation, rescaling, standardisation or categorisation of continuous predictors) |
| **Sample size** | Number of patients (i.e., number of patients in development and evaluation sets) |
|  | Number and proportion of events (i.e., number and proportion of events in development and evaluation sets) |
|  | Number of events per candidate predictor (i.e., number of events in relation to the number of candidate predictors in development set) |
|  | Justification of sample size (e.g., available/existing data, power, time interval, events per predictor) |
| **Missing data** | Handling of missing data (e.g., omission, imputation [e.g., mean, median, multiple imputation], or other methods) |
|  | Presentation of missing data (e.g., number of patients with any missing value [outcome or candidate predictors], number of patients with missing values for each candidate predictor) |
| **Analysis methods** | Supervised ML/AI method(s) (e.g., SVM, tree-based method [decision tree, gradient boosting machine, XGBoosting, random forest], neural networks) |
|  | Method for selection of predictors before modelling (a priori) (e.g., all candidate predictors, pre-selection based on univariable association with the outcome) |
|  | Method for selection of predictors during modelling (e.g., all candidate predictors, backward or forward selection, model-embedded selection, penalisation) |
|  | Method for dimensionality reduction (e.g., principal component analysis, factor analysis) |
|  | Method to handle class imbalance (e.g., resampling, undersampling, oversampling, class weighting) |
|  | Hyperparameter tuning strategy (e.g., grid search, random search, Bayesian optimization) |
|  | Method(s) of model evaluation (i.e., no evaluation with apparent performance, internal evaluation [e.g., data split, bootstrapping, cross-evaluation], external evaluation [e.g., chronological, geographical]) |
|  | Model adjustment or update after evaluation (i.e., updating/adjustment of a model in case of poor performance in evaluation) |
|  | Methods for calculation of predictions (e.g., formula, code, object, API) |
| **Performance** | Discrimination measures with confidence intervals (e.g., area under the curve, c-statistic, d-statistic) |
|  | Classification measures with confidence intervals and cut-points (e.g., sensitivity, specificity, predictive values) |
|  | Calibration measures with confidence intervals (e.g., O:E ratio, calibration slope, calibration intercept, calibration plot, Hosmer-Lemeshow statistic) |
|  | Additional presentation of the models (e.g., sum score, nomogram, score chart, predictions for specific risk subgroups) |
| **Model presentation and interpretation** | Number of final models |
|  | Number of final predictors selected in models |
|  | Type of final predictors selected in models |
|  | Importance of predictors in final models (e.g., weights, coefficients, mean decrease in accuracy) |
|  | Model generalizability/transportability discussion (i.e., discussion on study limitations [e.g., non-representative sample, sample size, overfitting, missing data] and model applicability) |
|  | Model usability in current care (i.e., discussion on intended and future use of the model) |
| **Open science practices** | Study protocol availability and registration |
|  | Data availability |
|  | Code availability |

API: Application Programming Interface; BSI: Bloodstream infection; CONSORT: CONsolidated Standards Of Reporting Trials; ECDC: European Centre for Disease prevention and Control; EMR: Electronic Medical Record; HAI: Healthcare-Associated Infection; ICU: Intensive Care Unit; ML/AI: Machine Learning/Artificial Intelligence; NHSN: National Healthcare Safety Network; O:E ratio: observed expected ratio; STARD: STAndards for the Reporting of Diagnostic accuracy studies; STROBE: STrengthening the Reporting of OBservational studies in Epidemiology; SVM: Support Vector Machine; TRIPOD: Transparent Reporting of multivariable prediction models for Individual Prognosis Or Diagnosis; UTI: Urinary Tract Infection; XGBoosting: eXtreme Gradient Boosting.
